# Supplementary material for: NEK7 regulates dendrite morphogenesis in neurons via Eg5-dependent microtubule stabilization
Source: Nat Commun. 2018 Jun 13;9:2330. doi: 10.1038/s41467-018-04706-7 (PMC5997995; doi:10.1038/s41467-018-04706-7)
Supplement: Supplementary file 1 — Supplementary Information [file 41467_2018_4706_MOESM1_ESM.pdf]

Supplementary Information

NEK7 regulates dendrite morphogenesis in neurons via Eg5-dependent microtubule stabilization.

Freixo et al.

Primer Name	Sequence
Nek7_F_FseI	TTTTTGGCCGGCCAATGGATGAACAATCACAAGGA
Nek7_R_AscI	TTTTTCGCGCCCCTTAGGTGCTTGCGGTACATG
Nek7_shres_F	GTAATTAAATACTATGCCTCCTTTATCGAAGACAATGAGCTGAAC
Nek7_shres_R	GTTCAGCTCATTGTCTTCGATAAAGGAGGCATAGTATTTAATTAC
Eg5_F_XhoI	GGACTCAGATCTCGAGATGGCGTCGCAGCCAAAT
Eg5_R_EcoRI	GTCGACTGCAGAATTCGAAGGTTGATCTGGGCTC
Eg5_F_FseI	TTTTTGGCCGGCCAATGGCGTCGCAGCCAAATTCG
Eg5_R_AscI	TTTTTGGCGCGCCTTAAAGGTTGATCTGGGCTCG
Nek7_F_Y97A	CCAAATGTAATTAAAGCCTATGCATCATTCATTG
Nek7_R_Y97A	CAATGAATGATGCATAGGCTTTAATTACATTTGG
Nek7_F_D179A	GTAAAACTTGGAGCTCTTGGGC
Nek7_R_D179A	GCCCAAGAGCTCCAAGTTTTAC
Eg5_F_S1033A	AACACACTGGAGAGGGCTAAAGTGGAAGAAACT
Eg5_R_S1033A	AGTTTCTTCCACTTTAGCCCTCTCCAGTGTGTT
Eg5_F_S1033D	AACACACTGGAGAGGGATAAAGTGGAAGAAACT
Eg5_R_S1033D	AGTTTCTTCCACTTTATCCCTCTCCAGTGTGTT
FLAG-Eg5_F_NheI	CGTCAGATGCGCTAGCATTGGATTATAACGATGAC
FLAG-Eg5_R_EcoRI	GTCCCTCGACGAATTCTTAAAGGTTGATCTGGGCTC
Nek7 gt/gt genotyping F	CTGAAGCAGGCCCTGAG
Nek7 gt/gt genotyping R	TCCATTAGCTCACAGTCATTACA

Supplementary Table 1. List of all primers used in this study.

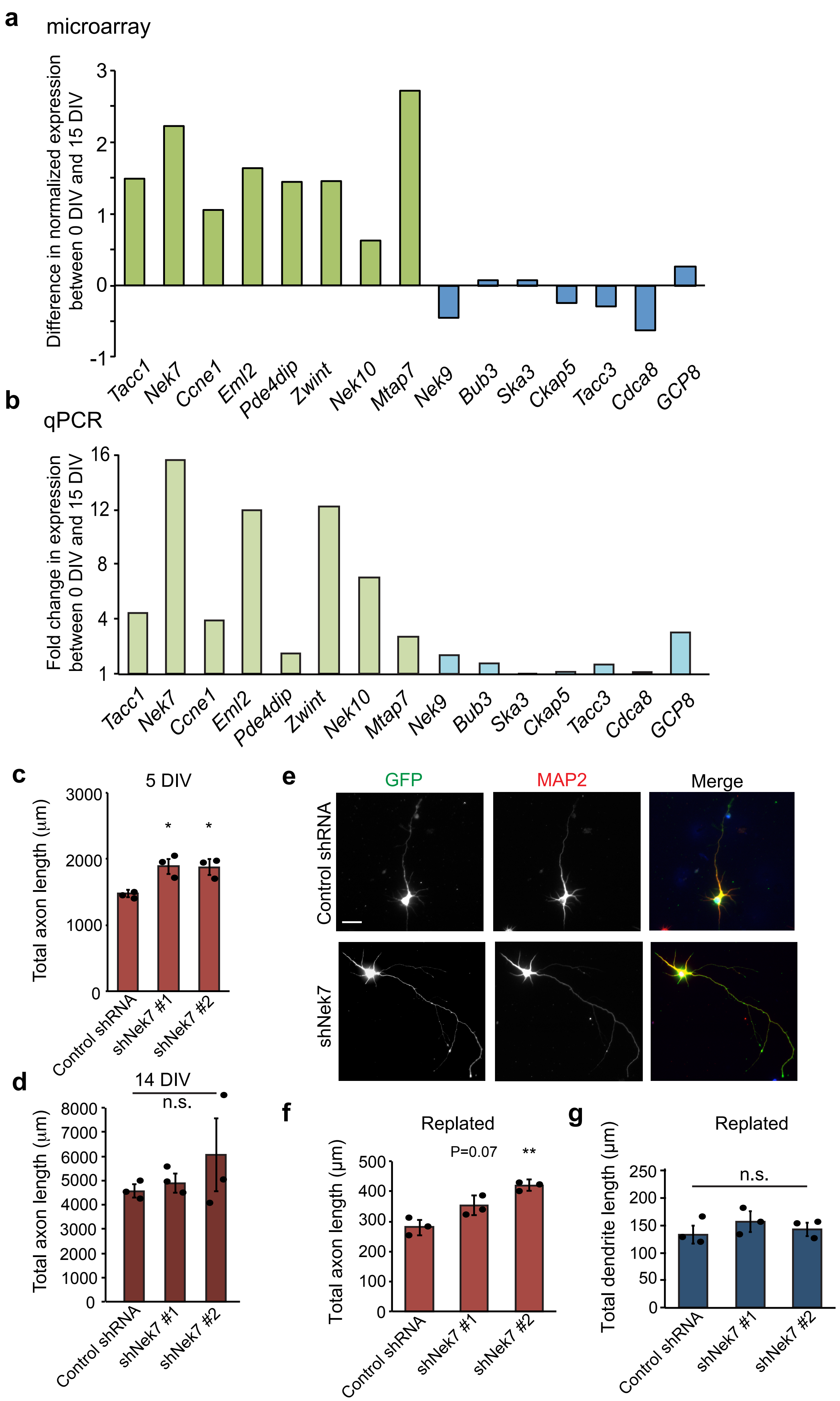

a
microarray
Difference in normalized expression between 0 DIV and 15 DIV
3
2
1
0
-1
Tacc1
Nek7
Ccne1
Eml2
Pde4dip
Zwint
Nek10
Mtap7
Nek9
Bub3
Ska3
Ckap5
Tacc3
Cdca8
GCP8
b
qPCR
Fold change in expression between 0 DIV and 15 DIV
16
12
8
4
1
Tacc1
Nek7
Ccne1
Eml2
Pde4dip
Zwint
Nek10
Mtap7
Nek9
Bub3
Ska3
Ckap5
Tacc3
Cdca8
GCP8
c
5 DIV
Total axon length (μm)
3000
2000
1000
0
*
*
Control shRNA
shNek7 #1
shNek7 #2
e
GFP
MAP2
Merge
Control shRNA
shNek7
d
14 DIV
n.s.
Total axon length (μm)
8000
7000
6000
5000
4000
3000
2000
1000
0
Control shRNA
shNek7 #1
shNek7 #2
f
Replated
P=0.07
**
Total axon length (μm)
500
400
300
200
100
0
Control shRNA
shNek7 #1
shNek7 #2
g
Replated
n.s.
Total dendrite length (μm)
250
200
150
100
50
0
Control shRNA
shNek7 #1
shNek7 #2

Supplementary Fig. 1. Expression analysis of candidate microtubule regulators and NEK7 knockdown phenotypes. (a) Microarray expression changes for short-listed genes in cultured neurons between 0DIV and 15DIV. **(b)** Expression changes for the genes in **a** were determined by quantitative real-time PCR. Reactions were performed in triplicate and normalized. Green columns represent "upregulated" genes, while blue columns represent "not differentially expressed" genes. **(c)** Mean total axon length in 5DIV neurons, transfected at 1DIV with plasmid expressing control or Nek7 shRNA, and GFP as marker. n=3 independent experiments. Total number of neurons: 90 (Control), 83 (shNek7 #1) and 63 (shNek7#2). * $P<0.05$, by two-tailed *t*-test. Error bars: s.e.m. **(d)** Mean total axon length in 14DIV neurons, transfected at 7DIV as in **c**. n=3 independent experiments. Total number of neurons: 39 (Control shRNA), 22 (shNek7 #1), 27 (shNek7 #2). n.s.: non-significant by two-tailed *t*-test**.** Error bars: s.e.m. **(e)** Immunofluorescence images of replated neurons, control or depleted of NEK7. GFP staining labels the whole cell while MAP2 preferentially stains soma and dendrites. Scale bar, 50µm. **(f)** Mean total axon length in neurons prepared as in **e**. n=3 independent experiments. Total number of neurons: 67 (Control shRNA), 58 (shNek7 #1), 46 (shNek7 #2). ** $P<0.01$ by two-tailed *t*-test**.** Error bars: s.e.m. **(g)** Mean total dendrite length in neurons prepared as in **e**. n=3 independent experiments. Total number of neurons: 23 (Control shRNA), 22 (shNek7 #1), 20 (shNek7 #2). n.s.: non-significant by two-tailed *t*-test**.** Error bars: s.e.m. Columns in all graphs show means and dot overlays individual data points.

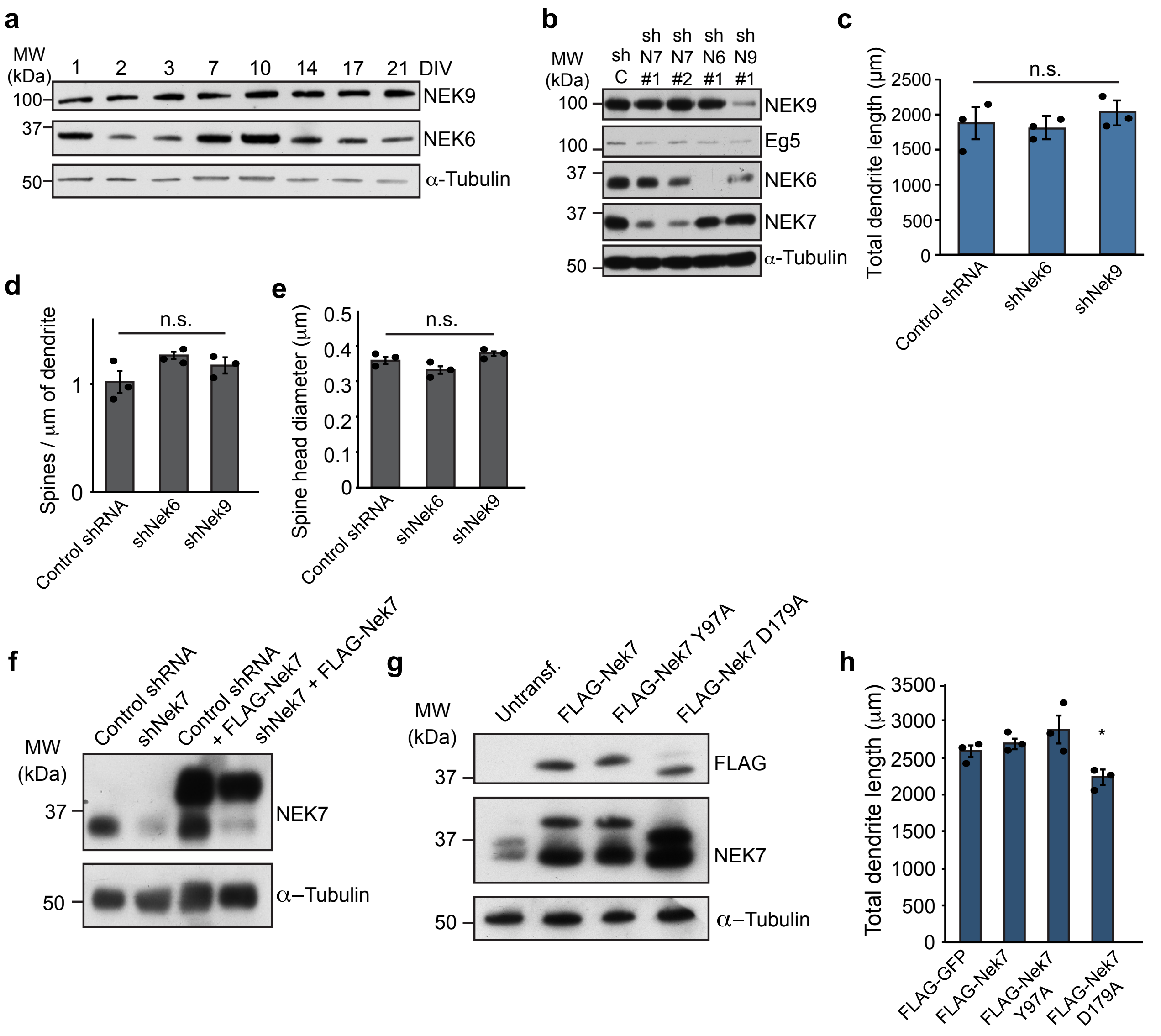

Supplementary Fig. 2. Phenotypes of NEK6 and NEK9 knockdown and expression of recombinant wild type and mutant NEK7. (a) Extracts of hippocampal cultures from 1DIV to 21DIV were immunoblotted against NEK6, NEK9, and α-tubulin as loading control. **(b)** 1DIV neurons were infected for 96h with virus expressing control shRNA, Nek9 shRNA, Nek6 shRNA or Nek7 shRNA, and cell extracts were immunoblotted with Eg5, NEK9, NEK6 and NEK7 antibodies. Detection of α-tubulin served as loading control. **(c)** 7DIV hippocampal neurons were co-transfected with a GFP plasmid and plasmid expressing control shRNA, Nek9 shRNA, or Nek6 shRNA. At 14DIV neurons were fixed, stained with GFP-antibody, and total dendrite length of GFP-positive neurons was quantified. n=3 independent experiments. Total number of neurons: 25 (Control shRNA), 26 (shNek6) and 26 (shNek9). n.s.: non-significant by two-tailed *t*-test**. (d)** Quantification of spine density in primary dendrites of neurons in the same experimental conditions as in **c**. n=3 independent experiments, 3-10 neurons per experiment, total number of spines analyzed per condition 665<n<1147. n.s.: non-significant, by two-tailed *t*-test. Error bars: s.e.m. **(e)** Quantification of spine head diameter in primary dendrites of neurons in the same experimental conditions as in **c**. Statistics as in **d. (f)** N2a cells were co-transfected

for 72h with plasmid expressing control shRNA or Nek7 shRNA, and FLAG-GFP or shRNA-resistant FLAG-NEK7. Probing of lysate by Western blotting with NEK7 antibody shows depletion of endogenous NEK7 but not of FLAG-NEK7 upon expression of Nek7 shRNA. α-Tubulin was used as loading control. **(g)** N2a cells were transfected for 72h with wild type FLAG-NEK7 or mutant FLAG-NEK7 (Y97A or D179A). Protein lysates were immunoblotted against NEK7 and FLAG. Western blot shows over-expression of FLAG-tagged NEK7 constructs relative to endogenous NEK7 in non-transfected N2a lysate. α-Tubulin was used as loading control. **(h)** Quantification of total dendrite length in 14DIV neurons expressing GFP, FLAG-NEK7 or NEK7 mutants as indicated. n=3 independent experiments. Total number of neurons: 53 (FLAG-GFP), 37 (FLAG-Nek7), 34 (FLAG-Nek7 Y97A) and 43 (FLAG-Nek7 D179A). *$P<0.05$ by two-tailed *t*-test**.** Error bars: s.e.m. Columns in all graphs show means and dot overlays individual data points.

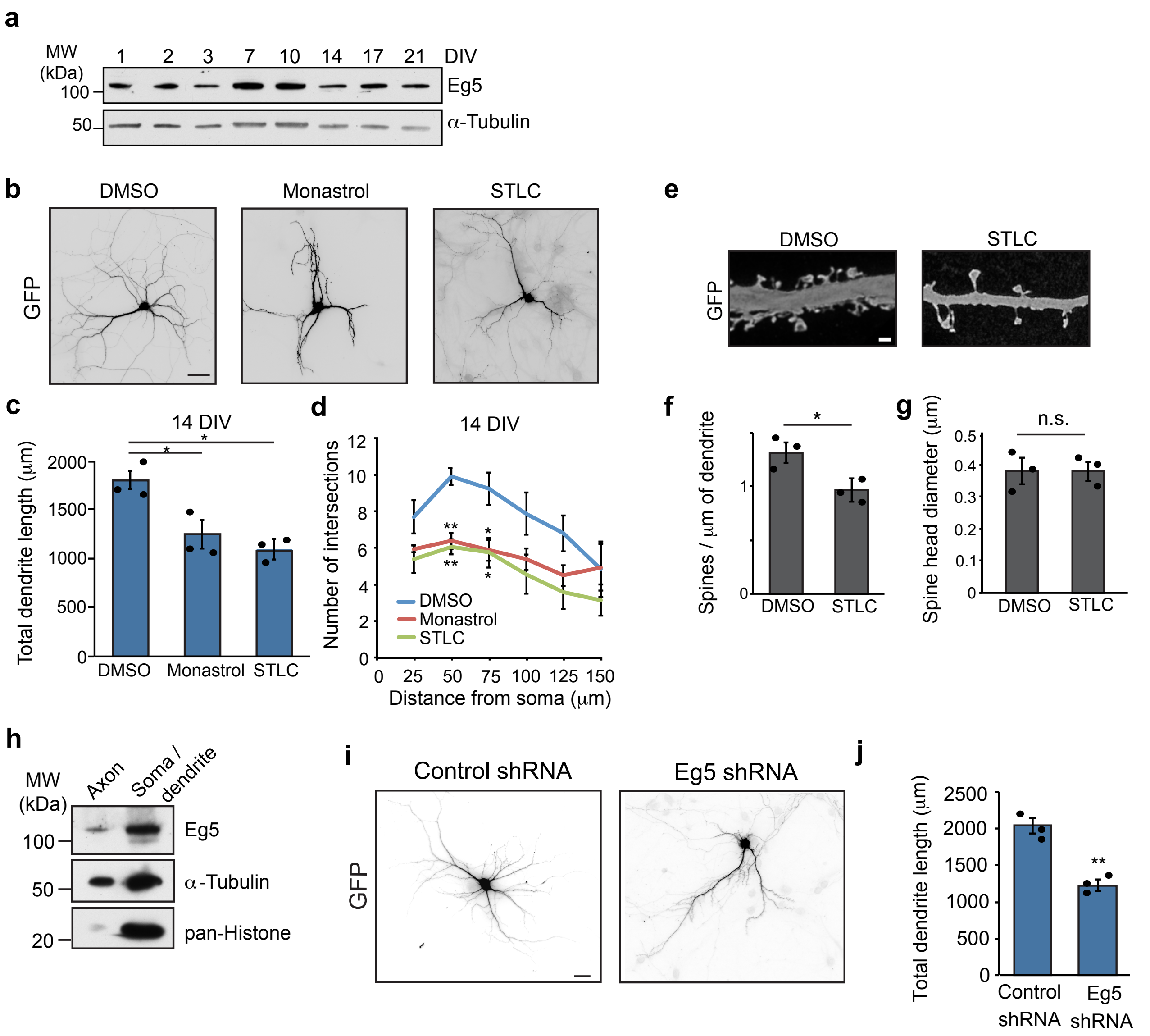

Supplementary Fig. 3. Phenotypes in neurons treated with Eg5 inhibitors or Eg5 shRNA. (a) Western blot detecting Eg5 in lysates from cultured neurons, from 1DIV to 21DIV, using α-tubulin as loading control. **(b)** Representative images of 14DIV neurons, transfected with GFP plasmid at 7DIV, and treated for the same period with either DMSO or the Eg5 inhibitors monastrol or STLC. Scale bar, 50μm. **(c)** Mean total dendrite length of 14DIV neurons in the experimental conditions shown in **b**. n=3 independent experiments, total number of neurons - 53 (DMSO), 58 (monastrol) and 40 (STLC). * $P<0.05$, by two-tailed *t*-test. Error bars: s.e.m. **(d)** Sholl analysis of neurons prepared as in **b**. Statistics as in **c**. **(e)** Representative images of spines in primary dendrites, from GFP-expressing neurons treated with STLC or DMSO as control. Scale bar, 1 μm. **(f)** Mean density of spines as shown in **e**. n=3 independent experiments**,** 7 to 14 neurons per experiment, total number of spines analyzed per condition: 946 (DMSO), 665 (STLC). n.s: non-significant, * $P<0.05$, by two-tailed *t*- test. Error bars: s.e.m. **(g)** Mean spine head diameter. Neurons were prepared as in **e** and statistics were as in **f**. **(h)** Lysates of somato-dendritic and axonal compartments of hippocampal neurons at 9DIV were

immunoblotted against Eg5 and pan-histone, using α-tubulin as loading control. **(i)** Neurons were transfected at 7DIV with either plasmid encoding control shRNA or plasmid encoding Eg5 shRNA, fixed at 14DIV and stained with GFP. **(j)** Mean total dendrite length of neurons as in a. n = 3 experiments, 32 neurons (Control shRNA) and 29 neurons (Eg5 shRNA). Error bars: s.e.m. ** P<0.01, by two-tailed *t*-test. Columns in all graphs show means and dot overlays individual data points.

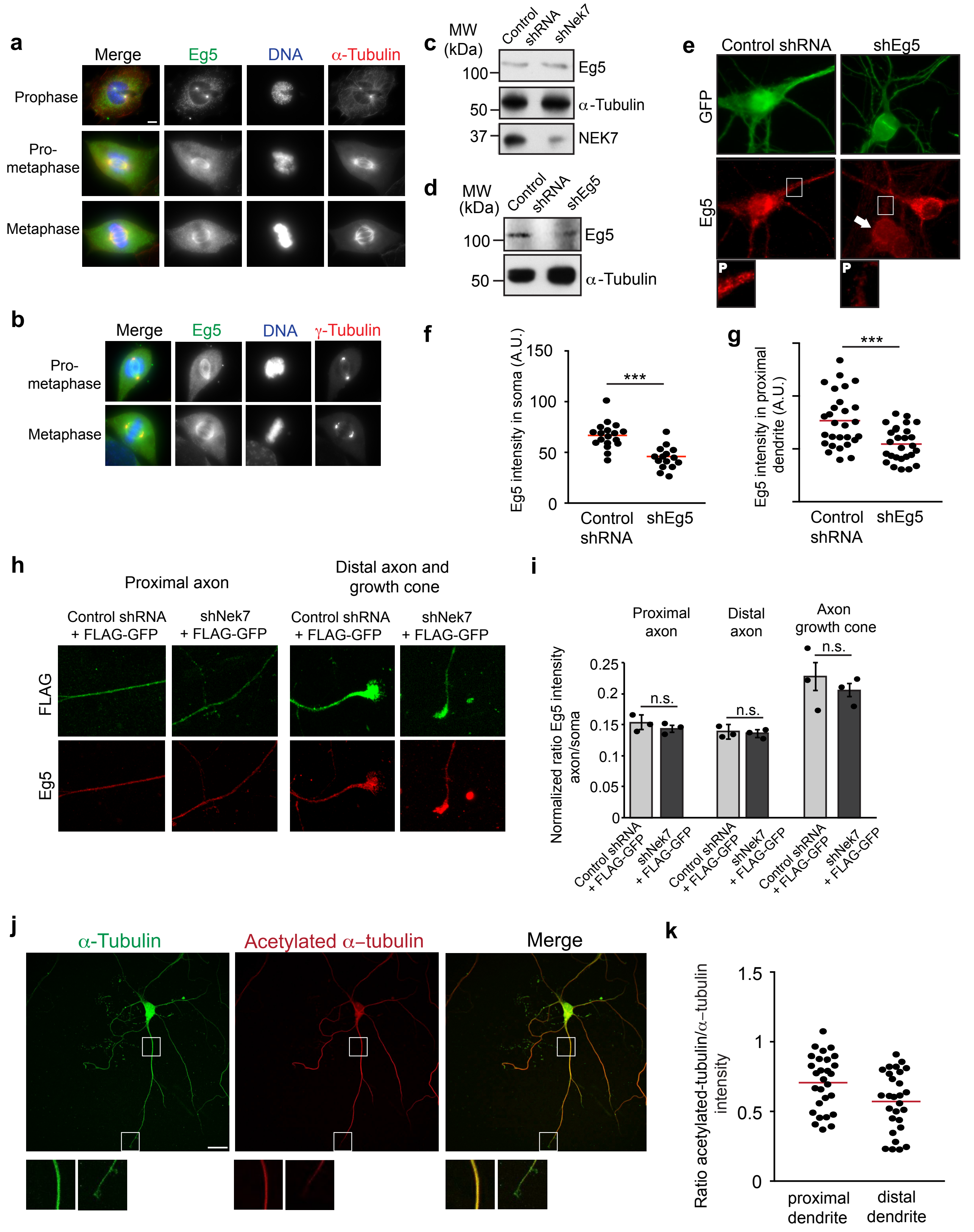

Supplementary Fig. 4. Specificity of Eg5 staining in neurons and quantification of acetylated microtubule staining in distal dendrites. (a) MEFs were stained with Eg5 and α-tubulin antibodies, and DAPI to label DNA. **(b)** MEFs were stained with Eg5 and γ-tubulin antibodies, and DAPI to label DNA. Scale bar, 10 µm. **(c)** Lysates of hippocampal neurons infected with lentivirus as indicated at 7DIV were analyzed at 14DIV by blotting

against Eg5 and NEK7, using α-tubulin as loading control. **(d)** Neuro2a cells were transfected as indicated and selected for 72 hours with puromycin. Cell lysates were blotted against Eg5, using α-tubulin as loading control. **(e)** Neurons were transfected at 7DIV as indicated, fixed at 14DIV and stained with GFP and Eg5 antibodies. The white arrow points at an Eg5-depleted cell. A section of the proximal dendrite is shown as magnification. **(f)** Quantification of the Eg5 intensity in the soma of neurons as in **e**. **(g)** Quantification of the Eg5 intensity in ~50 μm segments of proximal dendrites of neurons as in **e** n=14 neurons for each shRNA**.** Red lines: mean values. n***P<0.001 by two-tailed *t*-test. **(h)** Neurons transfected with the indicated plasmids at 7DIV were stained with FLAG and Eg5 antibodies. Panels show proximal and distal axon regions including the growth cone. Scale bar, 25 μm. **(i)** Quantification of the Eg5 intensity in ~50 μm segments of different axon regions as in **h**. Eg5 Intensities were plotted relative to the intensities in the somas of the same cells. n=3 independent experiments. Columns show means and dot overlays individual data points. Total number of neurons: 21 (Control), 18 (Nek7 depleted)**.** n.s.: non-significant, by two-tailed *t*-test. Error bars: s.e.m. **(j)** Confocal images of 9DIV neurons stained with antibodies against α-tubulin and acetylated α-tubulin. Proximal and distal dendrite regions are shown as magnifications. Scale bar, 50 μm. **(k)** Quantification of the ratio of acetylated/total α-tubulin intensity in proximal and distal parts of dendrites. n = 28 dendrites, from 10 neurons. Red lines: mean values.

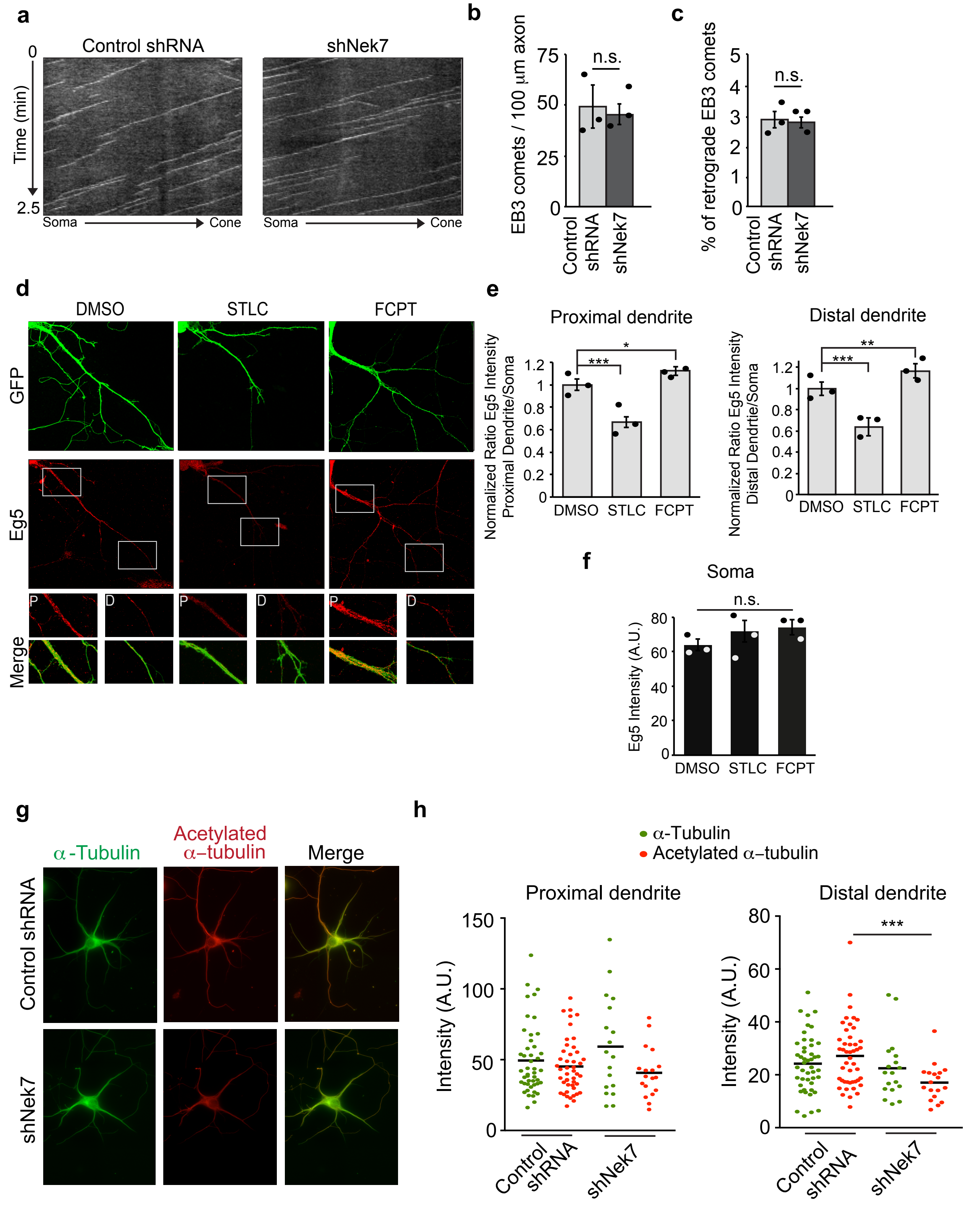

Supplementary Fig. 5. Lack of axonal phenotypes after NEK7 depletion, effect of inhibitors on Eg5 distribution, and destabilization of dendritic microtubules in NEK7 depleted neurons. (a) Representative kymographs of EB3 microtubule comets in axons of control or NEK7-depleted 9DIV neurons. **(b)** Quantification of EB3 comet density in axons of control or NEK7-depleted neurons. **(c)** Quantification of EB3 comet polarity in the same segments analyzed in (b). **(b,c)** n=35 axons, one per neuron, from 3 independent

cultures. Histograms show average ± S.E.M. n.s.: non- significant, by two-tailed *t*-test. **(d)** Neurons were transfected with a GFP plasmid at 7DIV, treated on the same day with either DMSO or the Eg5 inhibitors STLC or FCPT, and fixed at 14DIV and stained with GFP and Eg5 antibodies. Magnifications show Eg5 staining in proximal (P) and distal (D) dendrite regions. Scale bar, 50 µm. **(e)** Quantification of the Eg5 intensity in ~50 µm segments of proximal and distal dendrites of neurons as in **d**. Intensities were plotted relative to the intensities of Eg5 staining in the somas of the same cells. n=3 independent experiments. Total number of neurons: 30 (DMSO), 31 (STLC) and 29 (FCPT), 1 to 5 dendrites analyzed per neuron**.** n.s.: non-significant, *P<0.05, **P<0.01, ***P<0.001 by two-tailed *t*-test. Error bars: s.e.m. **(f)** Eg5 intensity in somas of neurons as in **d**. Statistics as in **e. (g)** Representative images of α-tubulin and acetylated α-tubulin staining in soma and dendrites of 9DIV neurons, transfected at 3DIV with plasmid expressing control shRNA or Nek7 shRNA. Scale bar, 25 µm. **(h)** Quantification of the raw intensity of α-tubulin (green dots) and acetylated α-tubulin (red dots) in proximal and distal dendrite regions of neurons prepared as in **g**. Each dot represents the mean intensity value for one cell. n=45 neurons (Control shRNA) and 18 neurons (shNek7) from 3 independent experiments. 2 to 4 dendrites were quantified per neuron. Black horizontal line: mean of the 3 experiments. *** P<0.001, by two-tailed *t*-test. Columns in all graphs show means and dot overlays individual data points.

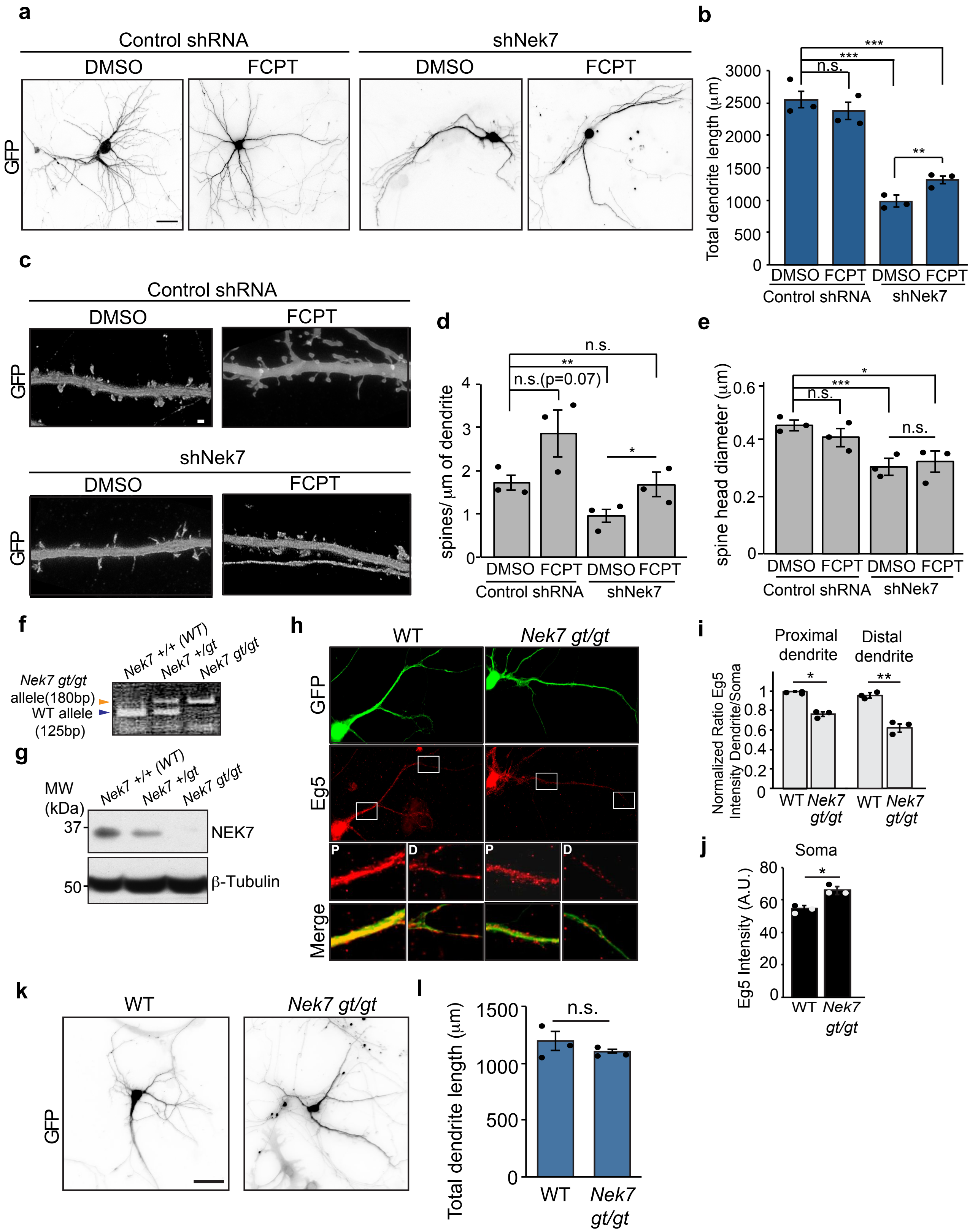

Supplementary Fig. 6. Effects of FCPT in NEK7-depleted neurons and analysis of neurons from *Nek7*$^{gt/gt}$ mice. (a) 14DIV neurons, co-transfected at 7DIV with Control or Nek7 shRNA and GFP plasmid, and treated with DMSO or FCPT. Scale bar, 50 μm. **(b)** Mean dendrite length at 14DIV in the conditions shown in **a**. n=3 independent experiments. Total number of neurons: 47 (Control shRNA and DMSO), 49 (Control

shRNA and FCPT), 24 (shNek7 and DMSO) and 36 (shNek7 and FCPT). n.s.: non-significant, ** P<0.01, *** P<0.001, by two-tailed *t*-test. Error bars: s.e.m. **(c)** Spines in primary dendrites of neurons as in **a**. Scale bar, 1 µm. **(d)** Mean density of spines as in **c**. n=3 independent experiments**.** Total number of spines: 684 (Control shRNA and DMSO), 758 (Control shRNA and FCPT), 256 (shNek7 and DMSO) and 437 (shNek7 and FCPT). n.s.: non-significant, *P<0.05, **P<0.01, *** P<0.001, by two- tailed *t*-test. Error bars: s.e.m. **(e)** Mean head diameters of spines as in **c**. Statistics as in **d**. **(f)** Genotyping of crosses of heterozygous *Nek7* gene trap mice. **(g)** e18.5 embryonic tissue lysates as indicated were probed by Western blotting with NEK7 antibodies. β-Tubulin was used as loading control. **(h)** Hippocampal cultures from wild type or $Nek7^{gt/gt}$ embryos were transfected with a GFP plasmid at 7DIV, fixed at 14DIV and stained with GFP and Eg5 antibodies. Magnifications show Eg5 staining in proximal (P) and distal (D) dendrites. **(i)** Mean Eg5 intensity in ~50 µm segments of proximal and distal dendrites as in **h**. n=3 wild type embryos and 3 $Nek7^{gt/gt}$ embryos. Total number of neurons: 28 (wild type) and 21 ($Nek7^{gt/gt}$). 1 to 4 dendrites per neuron. * P<0.05, **P<0.01 by two-tailed *t*-test. Error bars: s.e.m. **(j)** Eg5 intensity in somas of neurons as in **h**. Statistics as in **i. (k)** Images of wild type and $Nek7^{gt/gt}$ neurons as in **h,** fixed and stained with anti-GFP antibody **(l)** Mean dendrite length of neurons as in **k.** n.s.: non-significant, by two-tailed *t*-test. Error bars: s.e.m. Total of 35 (wild type) and 34 ($Nek7^{gt/gt}$) dendrites. Columns in all graphs show means and dot overlays individual data points.

Uncropped Western blots for Figure 1b:

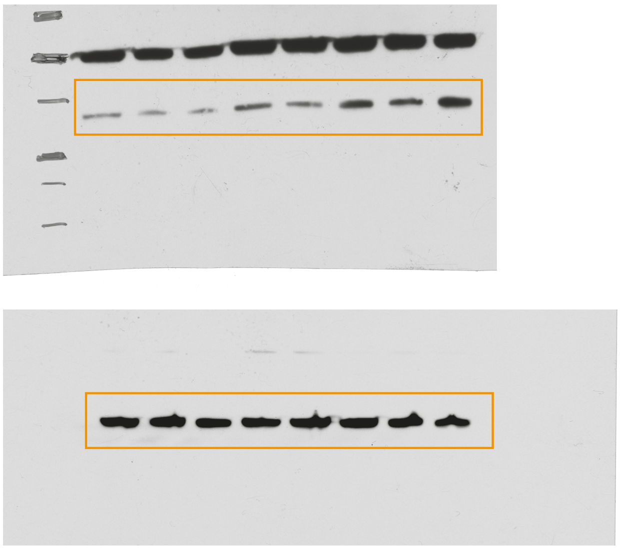

Uncropped Western blots for Figure 1d:

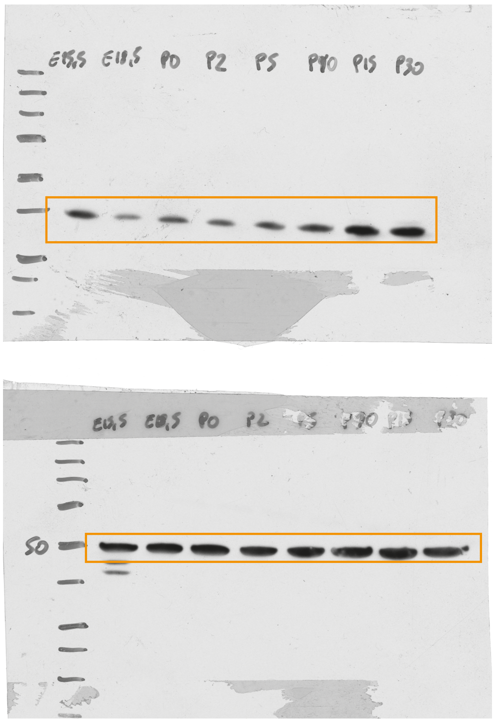

Uncropped Western blots for Figure 1f:

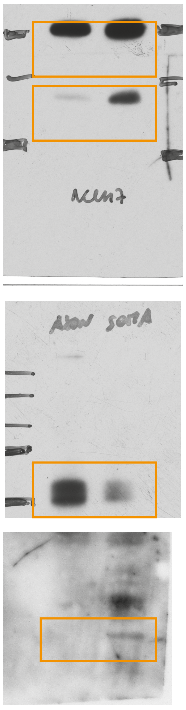

Uncropped Western blots for Figure 1g:

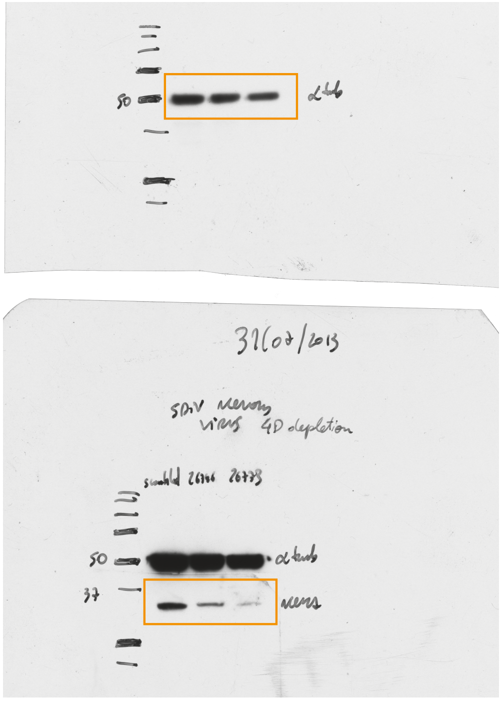

Uncropped Western blots for Figure 3a:

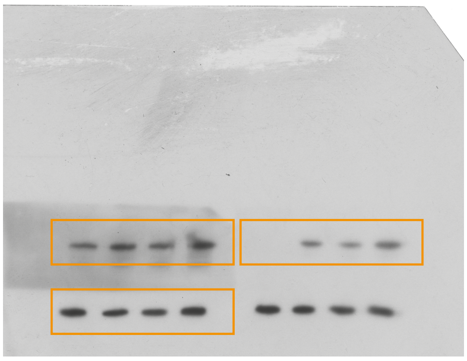

Supplementary Fig. 7. Uncropped Western blots Fig. 1 and Fig. 3.

Uncropped Western blots for Supplementary Figures 2a and 3a:

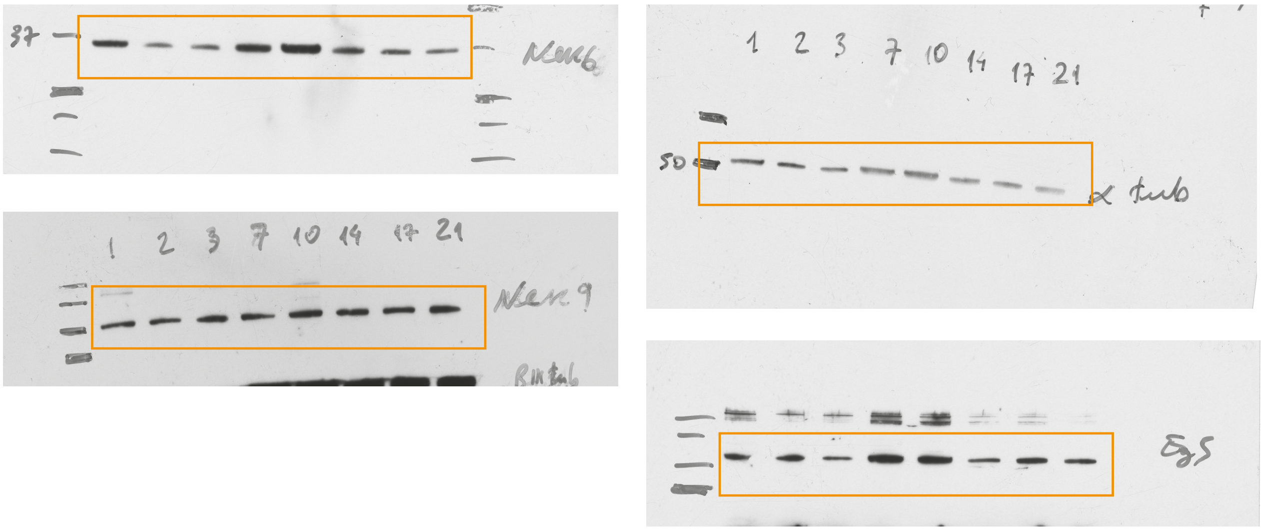

Uncropped Western blot for Supplementary Figure 2b:

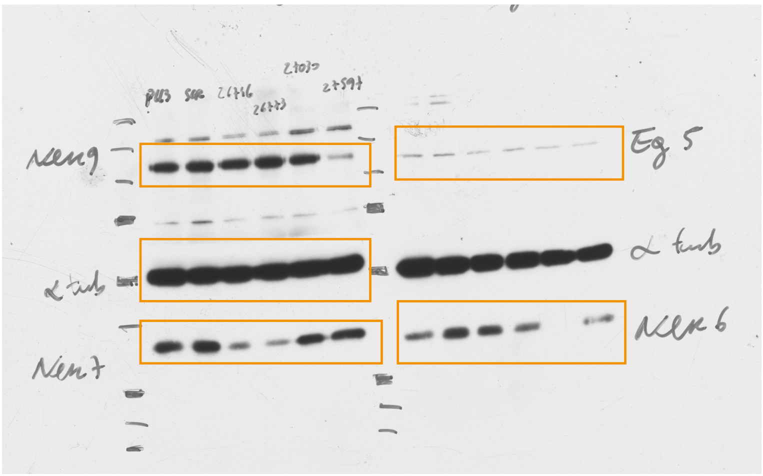

Uncropped Western blot for Supplementary Figure 2f:

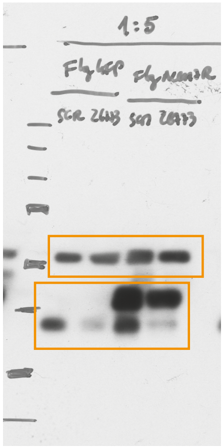

Uncropped Western blot for Supplementary Figure 2g:

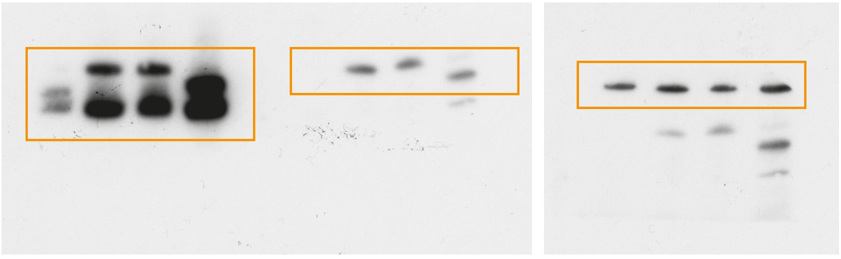

Uncropped Western blots for Supplementary Figure 3h:

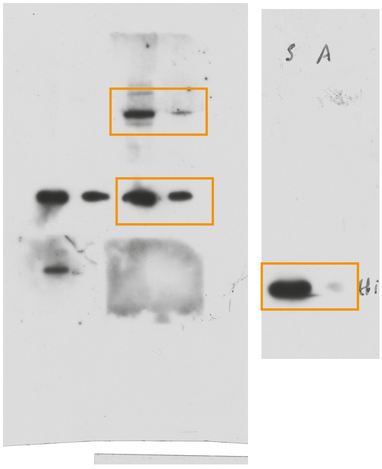

Supplementary Fig. 8. Uncropped Western blots Supplementary Fig. 2 and Supplementary Fig. 3.

Uncropped Western blot
for Supplementary Figure 4c:

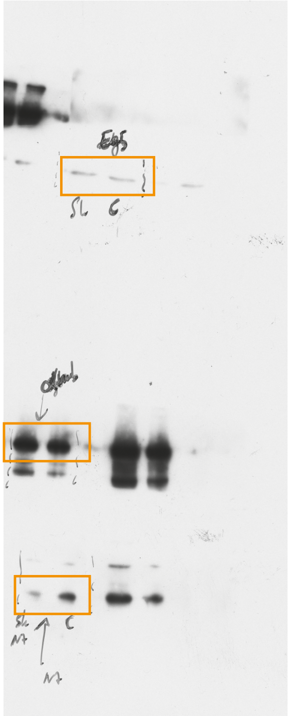

Uncropped Western blots for Supplementary Figure 4d:

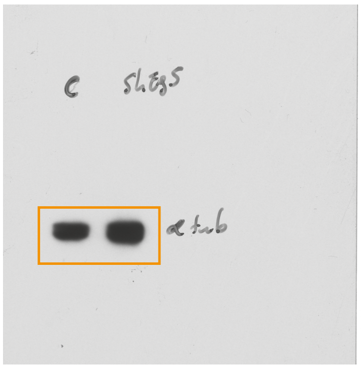

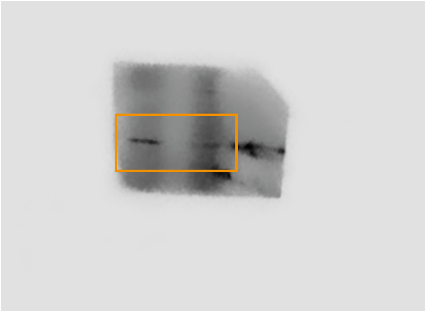

Uncropped Western blot for Supplementary Figure 6g:

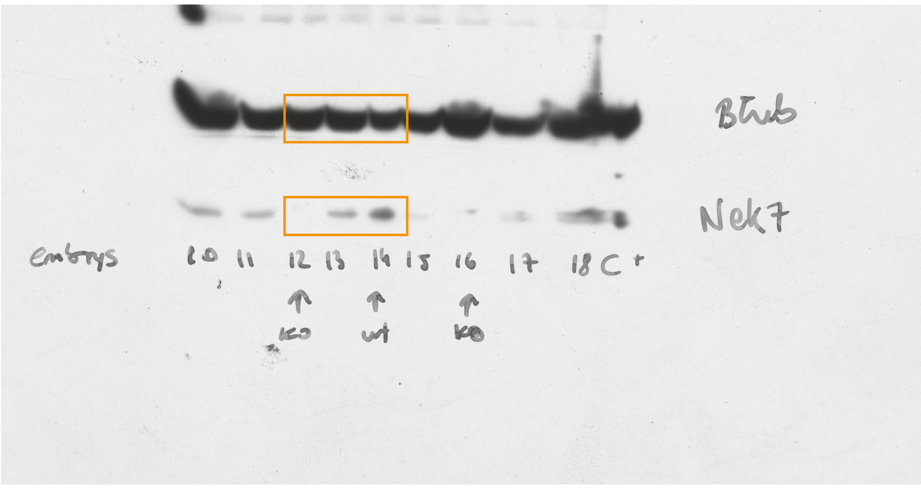

Supplementary Fig. 9. Uncropped Western blots Supplementary Fig. 4 and Supplementary Fig. 6.
