# Supplementary material for: NEK7 regulates dendrite morphogenesis in neurons via Eg5-dependent microtubule stabilization
Source: Nat Commun. 2018 Jun 13;9:2330. doi: 10.1038/s41467-018-04706-7 (PMC5997995; doi:10.1038/s41467-018-04706-7)
Supplement: Supplementary file 3 — Description of Additional Supplementary Files [file 41467_2018_4706_MOESM3_ESM.pdf]

Description of Additional Supplementary Files

File Name: **Supplementary Data 1**
Description: **Microarray expression levels of all genes.**

File Name: **Supplementary Data 2**
Description: **Microarray scaled expression levels of differentially expressed genes.**

File Name: **Supplementary Movie 1**
Description: **NEK7 depletion increases retrograde microtubule growth in distal dendrites.** Neurons were infected with the indicated lentivirus at 3DIV and transfected with EB3-Tomato at 8DIV, 24 hours before imaging the distal dendrites by time-lapse microscopy. The neuron soma is located towards the left.

File Name: **Supplementary Movie 2**
Description: **Absence of transport of short microtubules in dendrites.** Photobleaching of EOS-α−tubulin in 9DIV dendrites shows transport of a fluorescent vesicular cargo through the bleached area, but movement of tubulin polymers is not observed. Neurons were transfected at 3DIV with a plasmid expressing EOS-α-tubulin. Bleaching of the green EOS signal was performed and dendrites were imaged by time-lapse microscopy at 9DIV for a period of 15-20 minutes, with a 10 second interval between frames. The neuron soma is located at the left side of the videos.

File Name: **Supplementary Movie 3**
Description: **Stable binding of Eg5 to dendritic microtubules.** Neurons were transfected at 3DIV with plasmids expressing EOS, Eg5-EOS or EOS-α-tubulin. STLC was added 24h prior to imaging to neurons transfected with EOS-Eg5 plasmid. Dendrites were imaged by time-lapse microscopy at 9DIV. The signal of photoconverted EOS or EOS-tagged proteins is shown in the time-lapse videos. The neuron soma is located towards the left.
